# Clinical characteristics associated with GAD65 antibodies: a comparative study between high and low GAD65-ab cohorts

**DOI:** 10.3389/fneur.2026.1976777

**Published:** 2026-10-19

**Authors:** Paula Lombardo-Del Toro, Sònia Jaraba, Guillermo Hernández-Pérez, Velina Nedkova, Carlos Casasnovas, Serge Jauma-Classen, Laura Gonzalez-Mera, Roser Gómez-Llopico, Lydia García-Serrano, Jacint Sala-Padró, Francisco Morandeira, Mercè Falip

**Affiliations:** 1 Epilepsy Unit, Neurology Service, Hospital Universitario de Bellvitge, Hospitalet de LLobregat, Spain; 2 Neurology Service, Hospital Sant Joan Despí Moisès Broggi, Consorci Sanitari Integral, Sant Joan Despí, Spain; 3 Neurology Service, Hospital de Viladecans, Viladecans, Spain; 4 Neuromuscular Unit, Neurology Service, Hospital Universitario de Bellvitge, Hospitalet de LLobregat, Spain; 5 Movement Disorders Unit, Neurology Service, Hospital Universitario de Bellvitge, Hospitalet de LLobregat, Spain; 6 Central Laboratory Service, Immunology Department, Hospital Universitario de Bellvitge, Hospitalet de LLobregat, Spain

**Keywords:** cerebellar ataxia, epilepsy, glutamic acid decarboxylase antibodies, immune-mediated diabetes mellitus, limbic encephalitis, stiff-person syndrome

## Abstract

**Introduction:**

The frequency of neurological manifestations associated with serum GAD65 antibodies (NeuGAD) varies across studies, and the risk of neurological involvement in patients with high GAD65-ab titers remains unclear. This study aimed to determine the prevalence and clinical characteristics of neurological manifestations in patients with high (≥2,000 IU/mL) versus low (<2,000 IU/mL) serum GAD65-ab titers.

**Methods:**

We compared two cohorts: one cohort of 194 patients with diabetes mellitus (DM) diagnosed between 2000 and 2005 with positive low GAD65-ab titers, and a cohort of 150 patients with high GAD65-ab titers collected between 2013 and 2024. Groups were compared using the Mann–Whitney *U* test, chi-square/Fisher’s exact test, and multivariable logistic regression.

**Results:**

Among patients with high GAD65-ab titers, 32/150 (21.3%) had NeuGAD, most frequently presenting as epilepsy (13/32, 40.6%). Of the 128 patients with DM, 17 (13.3%) had NeuGAD. DM was present in 112/118 (94.1%) patients without NeuGAD, with frequencies varying by neurological syndrome without reaching significance. Age at NeuGAD onset was significantly lower in patients with DM than without DM (43.8 ± 13.5 vs. 55.4 ± 14.1 years, *p* = 0.03). None of the 194 low-titer patients developed NeuGAD (0%; 95% CI 0.0–1.9%).

**Conclusion:**

NeuGAD developed in 13.3% of DM patients with high GAD65-ab titers, while none of the low-titer patients developed NeuGAD, and it appeared at a younger age in patients with DM. We recommend serial GAD65-ab testing in patients with DM and high GAD65-ab titers, and prompt neurology referral when neurological symptoms arise, to allow earlier NeuGAD diagnosis.

## Introduction

Antibodies to glutamic acid decarboxylase (GAD-ab) were first detected in 1988. They are autoantibodies against the GAD enzyme that is mainly expressed in the central nervous system (CNS) and pancreatic β-cells. It catalyses the conversion of glutamate to GABA, the main inhibitory neurotransmitter in the CNS.

GAD65-ab are associated with autoimmune diseases such as diabetes mellitus (DM) and latent autoimmune diabetes in adults (LADA). Most patients with DM (80%) have low titers (<100 IU/mL) at onset. However, only patients with high serum titers (≥2,000 IU/mL) can present various neurological manifestations (NeuGAD), and the prevalence of cerebrospinal fluid (CSF) GAD65-ab positivity differs depending on the clinical presentation. These NeuGAD are frequently associated with other systemic autoimmune diseases such as thyroiditis, pernicious anemia, celiac disease, and vitiligo, as well as the presence of other related antibodies (1, 2). Treatment is based on immunomodulation; GAD65-ab titers tend to decrease after autoimmune treatment.

The neurological manifestations associated with GAD65-ab are well-defined diseases; nevertheless, they can be diagnostically challenging. These include:

### Stiff-person syndrome (SPS)

This is characterized by muscle spasms and progressive proximal rigidity. The presence of GAD65-ab, glycine receptor IgG or Amphiphysin IgG in serum and/or CSF is one of its diagnostic criteria, as updated in 2023 by Chia et al. (3)^.^

### Cerebellar ataxia

Clinically this may be characterized by vertigo, gait ataxia, nystagmus, dysarthria, dysphagia, and oculomotor dysfunction. Cranial magnetic resonance imaging (MRI) may show cerebellar atrophy although a normal MRI does not exclude the diagnosis. Diagnostic criteria for primary autoimmune cerebellar ataxia (PACA) include clinical symptoms, radiological criteria, and immunological criteria, including the presence of GAD65-ab (4).

### Autoimmune-associated epilepsy

Chronic epilepsy, often presenting as temporal lobe epilepsy, continuous focal epilepsy, or status epilepticus. This is associated with refractoriness to drug treatment and poor response to epilepsy surgery (5). It occurs without CSF inflammation but with intrathecal GAD65-ab synthesis in 70% of patients and normal MRI or progressive atrophy of one or both hippocampus (6).

### Limbic encephalitis

Characterized by subacute onset (<3 months) of behavioral disturbance, confusional syndrome, memory impairment, and seizures. MRI shows hyperintensities in T2 and FLAIR sequences at medial temporal lobes; CSF may show pleocytosis and protein elevation (7). It has recently been suggested that only a small proportion of patients with GAD65-ab meet the diagnostic criteria for limbic encephalitis, and this observation could indicate the presence of other, more pathologically relevant antibodies (8).

### Progressive encephalomyelitis with rigidity and myoclonus syndrome (PERMS)

Syndrome characterized by muscle rigidity, spasms, myoclonus, and brainstem dysfunction with oculomotor abnormalities, dysphagia, gait ataxia, prominent autonomic involvement, and decreased level of consciousness. It is considered the most severe presentation of the stiff-person spectrum. While anti-GlyR antibodies have a direct pathogenic role, GAD65-ab related cases are uncommon, and their role is considered less important than in SPS (9).

The association of GAD65-ab with cancer is rare; only 4–6% of patients with SPS and positive GAD65-ab have associated malignancies such as thymomas and breast cancer. These associations are, however, disputed (2, 8).

The percentage of patients with these neurological manifestations and GAD65-ab positivity varies from series to series. However, how many patients with high GAD65-ab titers develop NeuGAD and the timing of symptoms onset remain unknown.

The aim of the study was:

1. To determine the prevalence of neurological manifestations in patients with high GAD65-ab titers (≥2,000 IU/mL) in serum and to describe their clinical characteristics and their association with other autoimmune diseases.
2. To determine the prevalence of neurological manifestations in patients with low GAD65-ab titers (<2,000 IU/mL) in serum and to describe their clinical characteristics and their association with other autoimmune diseases.
3. To compare clinical and immunological characteristics of patients with high and low GAD65-ab.

## Methods

### Study design

We conducted a retrospective observational study. Two cohorts were analyzed and compared: (1) The database of the Diabetes Unit of the Hospital Universitari de Bellvitge (HUB) including all consecutive patients with a DM onset between 2000 and 2005 with positive GAD65-ab. (2) The database of the Immunology Department of the HUB including all patients with high GAD65-ab titers ≥2,000 IU/mL obtained in the laboratory between 2013 and 2024. Both databases were reviewed in 2024–2025 and the first database was reviewed in 2015 to search for patients with hypoglycemia episodes (10). Patients for whom insufficient information was available from shared medical records were excluded.

Clinical data were collected from shared clinical history. Epidemiological variables (age, sex), analytical variables (serum and CSF GAD65-ab titers, intrathecal synthesis (IS), CSF characteristics, oligoclonal bands (OCB), cellularity, proteins levels, and presence of other antibodies) and clinical variables (neurological manifestations, disease duration, and coexistence of other autoimmune diseases) were analyzed. GAD65-ab titers were not assessed during follow-up in patients without NeuGAD.

Patients from the two databases were initially classified into two broad groups, those without NeuGAD and those with NeuGAD. Patients with NeuGAD were classified into the following groups: SPS according to the diagnostic criteria (3), PERMS, cerebellar ataxia (4), autoimmune-associated epilepsy, limbic encephalitis according to the criteria of Graus et al. (7), or overlap symptoms, which is defined as neurological manifestations that comply with more than one neurological syndrome known to be associated with GAD65-ab and that cannot be explained by a more plausible diagnostic alternative.

Patient records were reviewed and classified by PL and MF. In case of diagnostic doubts, the records were also reviewed by SJ and VN.

The study was approved by the Ethics Committee of the HUB (PR189/24). The study was carried out in accordance with the principles of the Declaration of Helsinki. Confidential patient information was handled under the regulations of Spanish law (Organic Law 3/2018).

### Immunological analysis

GAD65-abs were determined in both serum and CSF. In the DM cohort, GAD65-ab were measured using a commercial ELISA (Henning, Berlin, Germany). In the high titers cohort, measurements were performed using an ELISA (RSR Ltd., Cardiff, UK), following the manufacturer’s instructions. A value greater than 5.0 IU/mL was considered positive both in serum and CSF. The upper limit of quantification for both assays was 2,000 IU/mL. Samples exceeding this threshold were reanalyzed after appropriate dilution to determine the exact concentration. When dilution could not be performed, the value of 2,000 IU/mL was assigned for statistical analysis.

In patients with high GAD65-ab and clinical suspicion of neurological disease another technique was used: commercial Immunoblot (Euroline PNS 12 Ag Test, Euroimmun, Germany), and when both techniques were positive, it was confirmed in our reference laboratory at Hospital Clinic.

To account for the IS of GAD65-ab, the GAD65-ab IgG index was calculated as described elsewhere (11). Values of GAD65-ab IgG index >0.7 were considered as positive. OCB were detected by isoelectric focusing using the Hydrasys Focusing System (Sebia Hispania, Barcelona, Spain) according to the manufacturer’s recommendations. The presence of OCB was considered positive when intrathecal immunoglobulin synthesis was observed.

### Statistics

A descriptive analysis of both qualitative and quantitative variables was performed. Qualitative variables are presented as absolute frequencies and percentages. All quantitative variables were assessed for normality (Shapiro–Wilk and Kolmogorov–Smirnov tests). Variables following a normal distribution in both groups were expressed as mean ± standard deviation and compared using Student’s *t*-test. GAD65-ab titers at onset and at follow-up, as well as age and age at DM onset in the low-titer group, did not meet normality assumptions; these variables were therefore expressed as median (interquartile range) in both groups and compared using the Mann–Whitney *U* test. Comparisons between groups for qualitative variables were performed using the chi-square test, or Fisher’s exact test when expected cell counts were below 5.

Multivariable analysis was performed using binary logistic regression, with GAD65-ab titer group (high vs. low titers; reference: low titers) as the dependent variable. Candidate variables were selected *a priori*: age, sex, DM, NeuGAD, number of other autoimmune diseases (categorical), hypothyroidism, thyroid disease, vitiligo, celiac disease, psoriasis, and atrophic gastritis. DM, NeuGAD, and the number of autoimmune diseases could not be estimated because of complete separation, as these variables had zero or near-zero frequency in the low-titer group; they were excluded from the final model and are reported descriptively in Table 1. The final estimable model included 9 variables. Age at DM onset was not included in this model because of collinearity with age and because it is only available for patients with DM. Statistical analyses were conducted using SPSS version 23 (IBM Corp.).

**Table 1:** Clinical characteristics of low and high GAD65-ab titers cohorts.

Characteristics	Low titers (<2,000 IU/mL)	High titers (≥2,000 IU/mL)	*p*-value univariate/*p* multivariate	OR
Patients (N)	194	150		
Gender: male/female male %	125/69 (64.5%)	60/90 (40.0)%	<0.001/0.001	0.363 (0.218–0.606)^a^
Age yr.; median (IQR)	48 (43–53.5)	54 (42–64)	0.001/0.219	1.014 (0.992–1.036)^b^
DM *N* (%)	194 (100%)	128 (85.3%)	<0.001	Not estimable^c^
NeuGAD	0(0%)	32(21.3%)	<0.001	Not estimable^c^
Age at DM debut year; median (IQC)	26 (22–33)	43 (32–53)	<0.001/—	Not included in this model^d^
Patients with only 1 other autoimmune diseases	37 (19.1%)	37 (25%)	0.187/—	Not estimable^c^
One or more other autoimmune diseases	46 (23.7%)	54 (36.5%)	0.010/—	Not estimable^c^
Thyroid diseases; *n* (%)	23 (11.1%)	32 (20.9%)	0.037/0.485	0.628 (0.170–2.317)
Hypothyroidism; *n* (%)	18 (8.2%)	23 (15.5%)	0.058/0.330	1.967 (0.504–7.682)
Vitiligo; *n* (%)	6 (3%)	7 (4.7%)	0.186/0.711	1.419 (0.223–9.036)
Psoriasis; *n* (%)	6 (3%)	4 (2.6%)	0.896/0.883	1.138 (0.202–6.410)
Atrophic gastritis; *n* (%)	3 (1.5%)	9 (6.1%)	0.036/0.195	0.322 (0.058–1.789)
Celiac disease; *n* (%)	2 (1%)	6 (4.1%)	0.086/0.259	0.332 (0.049–2.251)

a Reference category: female. OR from the multivariable model.

b Per year; equivalent to OR ≈ 1.15 per decade.

c DM, NeuGAD, and the number of other autoimmune diseases (entered as a categorical variable, 1/2/3 vs. 0) could not be estimated in the multivariable model because of complete separation (zero or near-zero cell counts in the low-titer group); frequencies are reported descriptively only.

d Age at DM onset was excluded from the main multivariable model because of collinearity with age and because it is only available for patients with DM.

## Results

### Patients with high GAD65-ab titers

From the laboratory database 148 patients with GAD65-ab titers ≥2,000 IU/mL were identified and included. Three patients from the DM database had titers ≥2,000 IU/m; one was duplicated, so two were added, resulting in a total cohort of 150 patients with high GAD65-ab levels (Figure 1). The mean age was 53.0 ± 15.97 years, and 90 (60%) were female. NeuGAD was present in 32 (21.3%) patients with the follow neurological syndromes: epilepsy 13 (8.6%), one with a previous history of encephalitis, SPS 10 (6.6%), overlap in 6 (4%), cerebellar ataxia 2 (1.33%) and ophthalmoparesis 1 (0.67%). One patient with PERMS and ophthalmoparesis had a malignant thymoma and tested positive for glycine-receptor, CV2, and Zic4 antibodies in addition to GAD65-ab. The clinical picture fulfilled criteria for a paraneoplastic neurological syndrome most likely associated with glycine-receptor antibodies; GAD65-ab positivity was therefore considered a non-contributory finding, and the patient was not classified as NeuGAD (Figures 1, 2).

**Figure 1:**
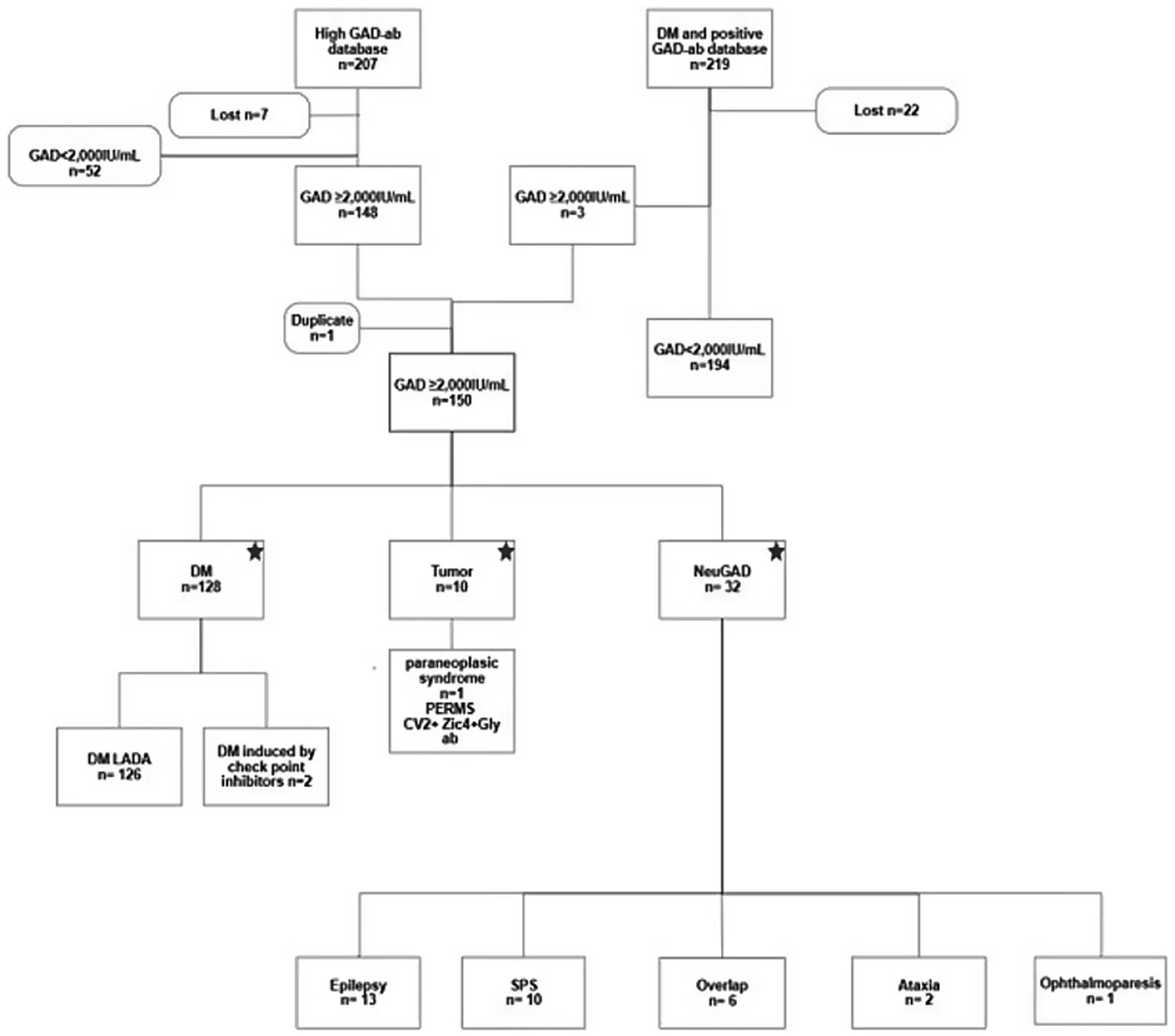
Flowchart of patients included in the study from the high GAD65-ab titers database from 2013 to 2024 year and the low GAD65-ab titers and DM database from 2000 to 2005. *The three boxes are not mutually exclusive.

**Figure 2:**
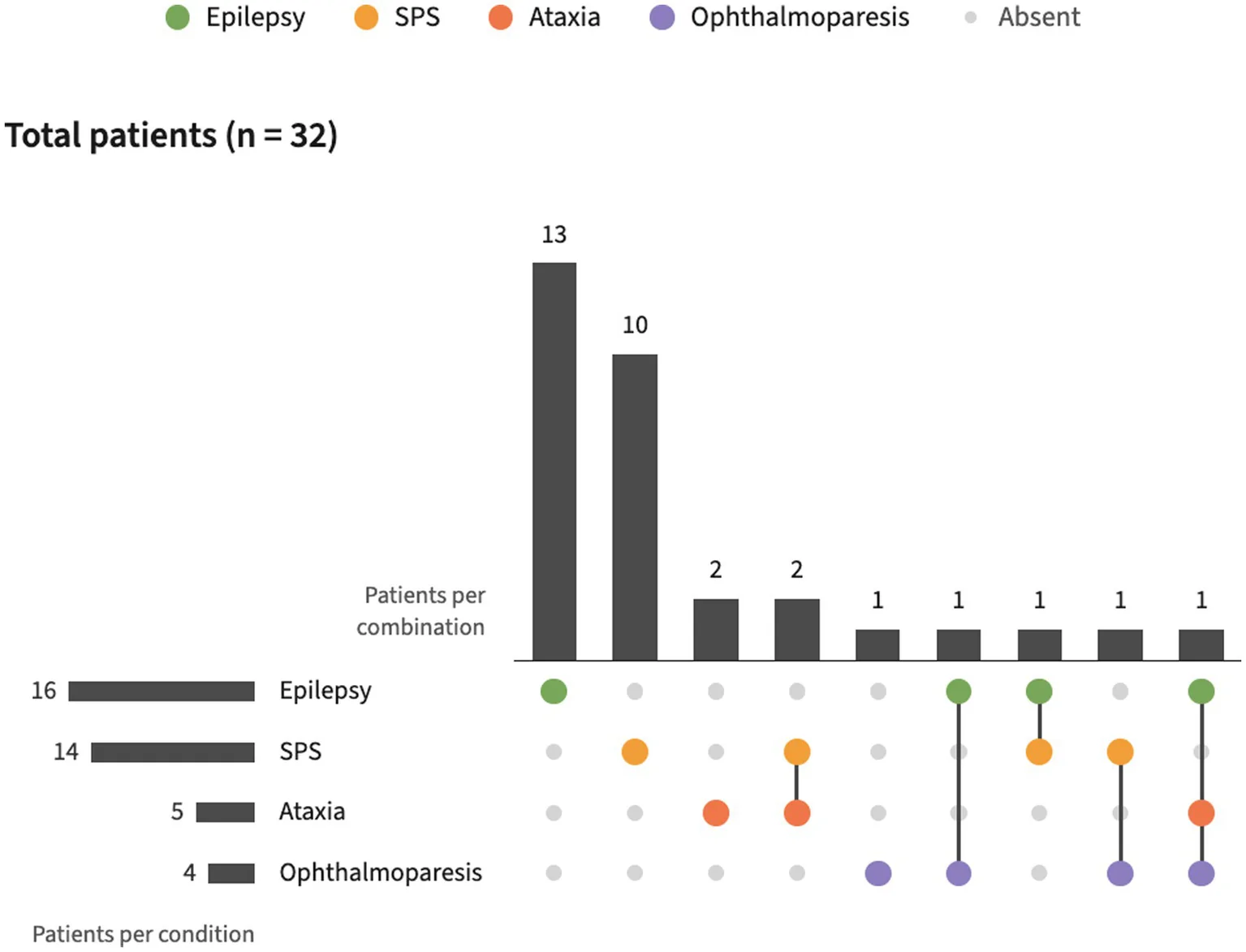
Upset plot of the different neurological GAD65-ab-diseases across the patients, showing the patients with overlap in more than one color.

DM was present in 128 patients (85.3%), including 126 (98.4%) with DM-LADA and 2 (1.6%) with immune checkpoint inhibitor-induced DM (pembrolizumab, ipilimumab, and nivolumab). Among DM patients, 17/128 (13.3%) had confirmed NeuGAD.

Tumors were identified in 10 (6.7%) patients, only one of whom had neurological manifestations, consisting of PERMS and ophthalmoparesis (see above) (Figure 1).

Sex-related differences were assessed for NeuGAD, DM, and age at onset. NeuGAD was present in 13/60 men (21.7%) and 19/90 women (21.1%), with no significant difference (OR 1.03, 95% CI 0.47–2.29; *p* = 0.935). DM was present in 54/60 men (90.0%) and 74/90 women (82.2%), again without a significant difference (prevalence ratio 1.09, 95% CI 0.96–1.24; OR 1.95, 95% CI 0.71–5.30; *p* = 0.187).

No significant differences were found in the age at DM onset in patients with NeuGAD (45.65 ± 17.75 years) compared to DM patients without NeuGAD (mean age of DM onset: 42.23 ± 14.19 years). On the other hand, the mean age at onset of NeuGAD was significantly lower in DM patients (43.8 ± 13.51 years compared to non-DM patients at 55.36 ± 14.1 years, *p* = 0.03).

Nearly all patients without neurological GAD65-ab diseases suffered DM (111/118, 94.1%) but the percentage of patients with DM differed among the different neurological GAD65-ab diseases (Figure 3). The majority of patients with epilepsy had DM (9/13, 69.2%); in contrast, DM was less frequent in patients with SPS (4/10, 40.0%) and in those with an overlap syndrome (2/6, 33.3%) (Figure 3). None of these differences was statistically significant.

**Figure 3:**
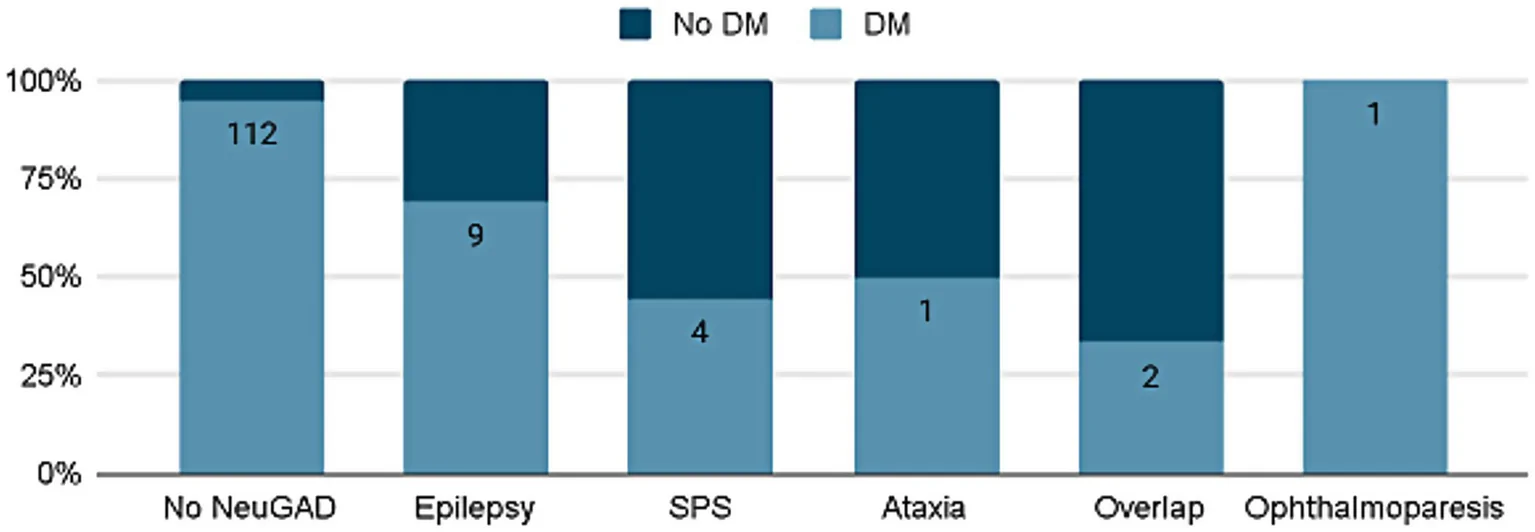
Bar chart showing the prevalence of DM among patients according to their different neurological disorders.

Out of the total cohort with high GAD65-ab titers (autoimmune disease data were missing for 2 patients), 54 (36.48%) had an autoimmune disease other than DM or NeuGAD (see Table 1). Of them, 37 patients (25%) had one additional autoimmune disease, 14 (9.5%) had two, and 3 (2%) had three. Having a higher number of autoimmune diseases did not imply a higher risk of developing NeuGAD, OR 0.7 (CI 0.37–1.34). No significant differences were found in NeuGAD prevalence according to the presence of associated autoimmune diseases. NeuGAD was numerically more frequent in patients with vitiligo 3/7 (42.9%) than in those without vitiligo 29/143 (20.3%), although this difference was not statistically significant (*p* = 0.17). Among NeuGAD patients, 3/32 (9.4%) had vitiligo.

Other autoantibodies were detected in 95 patients (63.3%), including 81/128 (63.3%) of those with DM and 18/32 (56.3%) of those with NeuGAD; these two subgroups overlap, as 17 patients had both DM and NeuGAD. The most frequently detected were anti-tyrosine phosphatase 2 antibodies (IA2) and anti-thyroid peroxidase antibodies (anti-TPO). Anti-TPO results were available for 136 of 150 patients (31 with NeuGAD, 105 without). Anti-TPO was observed in 9/31 (29.0%) of patients with NeuGAD, and in 18/105 (17.1%) of patients without NeuGAD. All IA2-positive patients (22/22, 100%) had DM, but only 2/22 (9.1%) of them had NeuGAD.

Patients with NeuGAD had significantly higher titers (mean rank 85.09 vs. 68.90; Mann–Whitney *U* = 1,389, *Z* = −2.475, *p* = 0.013) compared with patients without neurological involvement. Although the median was 2,000 IU/mL in both groups, the distribution in NeuGAD extended towards markedly higher values (third quartile 41,357 vs. 2,000 IU/mL; range 2,000–7,320,000 vs. 2,000–1,580,000 IU/mL).

CSF samples at onset were only available from 11 patients with high GAD65-ab in serum, 10 of them with NeuGAD and 1 without. GAD65-ab were detected in CSF in 8 patients using both ELISA and commercial ELISA assay; in two additional patients, positivity was identified by a single method (one ELISA, one commercial assay) and confirmed by our reference laboratory. OCB data were available for 7 patients, of whom 6 had OCB and these also had IS; one patient presented IS but not OCB. In the patient who had no NeuGAD, genetic testing revealed a pathogenic variant consistent with CANVAS and despite high serum GAD65-ab titers, GAD65-ab was not detected in CSF by ELISA confirmed by cell based assay and tissue based assay; therefore the patient was not considered to be NeuGAD affected (see Figure 4).

**Figure 4:**
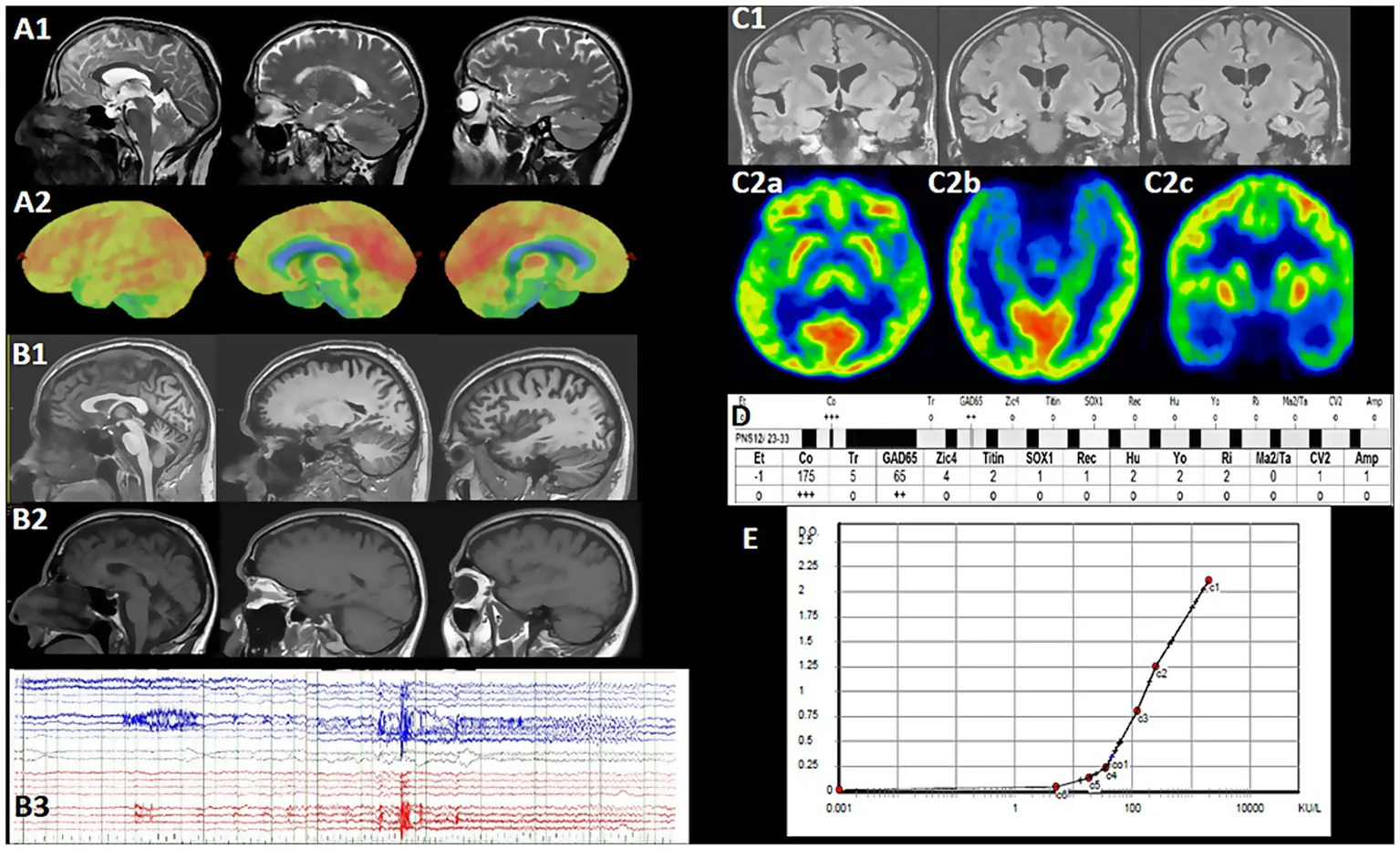
**(A)** Male patient with subacute ataxia and high GAD65-ab. **(A1)** Sagittal T2 MRI of the patient without cerebellum atrophy. **(A2)** FDG-PET of the same patient with slight hypometabolism in brain pons, both cerebellum hemispheres (predominantly in the lower margin), and the frontobasal cortex. **(B)** Male patient with ataxia and high GAD65-ab at onset with self-limited diplopia and 2 years later developing progressive ataxia; 10 years following onset he started suffering temporal lobe seizures. **(B1)** Sagital T1 MRI (2016) with vermis atrophy, and **(B2)** progression of vermis atrophy (2023). **(B3)** EEG showing a left temporal lobe seizure induced by hyperventilation **(C)** Patient with epilepsy and high GAD65-ab. **(C1)** Coronal FLAIR MRI sequences showing atrophy and increased signal in left hippocampal formation. **(C2)** FDG-PET. **(C2a)** axial slice showing left insular hypometabolism. **(C2b)** Axial slice showing bilateral anterior and mesial hypometabolism. **(C2c)** Coronal slices showing bilateral mesial temporal hypometabolism. **(D)** Profile of antineuronal antibodies by commercial immunoblot showing moderate positive (++) reactions to anti-GAD65 antibody; the positive control (Co) shows strong reactivity (+++), and the negative control (Et) is non-reactive, confirming assay validity. **(E)** Calibration curve for the quantitative determination of anti-GAD65 antibodies in serum by ELISA. This shows the relationship between optical density (OD) and the concentration of calibrators expressed as KU/L (equivalent to IU/mL) on a logarithmic scale. Points (C1: 2000; C2: 250; C3: 120, C4: 35; C5: 18; C6: 5).

### Patients with low GAD65-ab titers

Of 219 patients, medical records could be accessed for 197. All of them had DM and positive GAD65-ab, with high levels (≥2,000 IU/mL) in 3. Of these three patients, one had NeuGAD (epilepsy) and was already in the high-titer database; the other two, without NeuGAD, were added to the high-titer cohort (see above and Figure 1). Two patients (2/194, 1.0%) had intermediate GAD65-ab titers (500 to <2,000 IU/mL). One of them suffered a well-controlled epilepsy (non-lesional temporal lobe epilepsy) and the other suffered multiple sclerosis with a previous onset and suffered a DM debut while being treated with interferon beta; this patient was classified as drug-induced DM without NeuGAD. Neither of the two diseases was considered GAD65-ab related.

The median age was 43 years (48–53.5), and 125 (64.5%) were men. None of the 194 low-titer patients developed NeuGAD (0%; 95% CI 0–1.9%) in 2015 and in 2025. During follow-up, 16/194 (8.2%) patients with DM developed neurological diseases not related to GAD65-ab, the most frequent being polyneuropathy probably related to the DM glycemic control. Median serum titer level in patients with DM was 32 IU/mL (range 5–295). Other clinical characteristics of this cohort are reported in Table 1.

Patients with low and high GAD65-ab titers were compared using univariate and multivariate analyses. In the multivariable model, male sex was associated with lower odds of belonging to the high-titer group (OR 0.363, 95% CI 0.218–0.606, *p* < 0.001), while age was not independently associated with titer group (OR 1.014 per year, 95% CI 0.992–1.036, *p* = 0.219). None of the other autoimmune diseases assessed showed a statistically significant association in the multivariable model (Table 1).

## Discussion

In our cohort only 21.3% of patients with high GAD65-ab titers (≥2,000 IU/mL) had NeuGAD, a markedly lower proportion than previously reported. In the series reported by Saiz et al. (12), 82% of patients with high GAD65-ab titers had neurological disorders. This high proportion likely reflects the clinical context of that cohort, as GAD-ab testing was mainly requested for patients already referred from neurology or endocrinology clinics, resulting in a population enriched for neurological or endocrine disease. In contrast, GAD-ab testing is now performed routinely in most patients with adult-onset DM. Because our cohort was derived from a laboratory database rather than from patients previously referred to neurology clinics, it likely captures a broader, less selected patient population, which may explain the lower frequency of neurological involvement observed in our study. On the other hand, other studies have proposed that GAD65-ab titers >10,000 reflect higher specificity for the development of NeuGAD. In the cohort reported by Muñoz et al., 94% of patients with titers >10,000 presented with a classical GAD65-ab related neurological syndrome (13). We recognize that higher GAD65-ab titers increase diagnostic specificity. However, our study identified patients with epilepsy and GAD65-ab titers <10,000 IU/mL, indicating that using this threshold may reduce sensitivity, as suggested in a recent study (14). It is possible that the cut-off value should differ among various neurological disorders, but further studies are needed to define these points (14).

GAD65-ab titers at disease onset were higher in patients with NeuGAD, while the mean of titers at onset of neurological disease vary across studies, ranging from 74,700 IU/mL (13) to 720,000 IU/mL, and levels have been previously reported to be higher in patients with ataxia or limbic encephalitis (15). GAD65-ab titers at follow-up were available only for patients with neurological disease, as follow-up testing is not routinely performed by endocrinologists. These titers were lower at follow-up than at baseline, and remained positive in all cases after treatment, which is consistent with previous studies (16).

Only 1.5% of patients with DM had high GAD65-ab titers, and none of those with low GAD65-ab titers developed NeuGAD after 20–25 years of follow-up, lending support to the notion that high GAD65-ab titers constitute a risk factor for neurological manifestations. However, in isolation, they are insufficient to establish a definitive GAD65-driven autoimmune neurological disorder (9, 15, 24). Therefore, it may be beneficial to longitudinally monitor patients with DM and GAD65-ab titers ≥2,000 IU/mL, in order to identify those with increasing levels, as suggested in prior studies (16). This approach may facilitate earlier neurological evaluation, diagnosis, and treatment of NeuGAD, with the goal of modifying disease progression, improving clinical outcomes, and potentially preventing complications such as cerebellar atrophy in cerebellar ataxia and temporal lobe atrophy in epilepsy. In addition, it may contribute to better seizure control in drug-resistant epilepsy and help prevent cognitive impairment (6, 17, 18). Interestingly, a recent study reported increased neurofilament levels across the different neurological syndromes associated with GAD65-ab, with the highest concentrations observed in cerebellar ataxia and limbic encephalitis. Levels were higher at disease onset compared to follow-up, suggesting a higher neuronal destruction at the beginning of the disease. The study also revealed similar patterns of brain atrophy among different disease phenotypes, including evidence of cerebellar degeneration in patients without overt ataxia, highlighting the importance of early diagnosis (19).

The mean age at onset of neurological GAD65-ab disease was significantly lower in DM patients, therefore, DM was associated with earlier onset of NeuGAD. In our cohort, 53.1% of patients with neurological involvement had DM, which is consistent with previous reports (2, 12, 15). However, only 13.3% of DM patients with high titers developed NeuGAD whereas none of the patients with low titers developed NeuGAD. Other characteristics related to the development of NeuGAD are being female, the presence of other autoimmune diseases, such as thyroiditis, pernicious anemia, or vitiligo, and the presence of other autoantibodies such as anti-TPO, anti-thyroglobulin, antiparietal cells, and anti-gliadin antibodies (2). However, in our cohort, NeuGAD was not associated with sex, the number of other autoimmune diseases, vitiligo. The prevalence of vitiligo in patients with NeuGAD was 9.4%, which is notably lower than the 18.75% reported by Bai et al. (20). This discrepancy may be explained by the retrospective design of our study and the fact that patients were followed up by endocrinologists and neurologists, which could have led to an underreporting of dermatological conditions. Interestingly, all IA2-positive patients had DM; however, only 9.1% of IA2-positive patients exhibited NeuGAD. This result is consistent with a previous study published in 1997 which evaluated the specificity of IA2 and GAD65-ab in the diagnosis of DM. In this study, IA2 were detected in 56% of DM patients, while GAD65-ab were present in 68%. Notably, only 14% of patients with SPS were positive for IA2, compared to 89% who were GAD65-ab positive. Therefore, it was concluded that IA2 had a higher specificity for the diagnosis of DM than GAD65-ab (21).

It is well established that the development of autoimmune diseases is influenced by a combination of genetic predisposition and environmental factors that can act as triggers. This may explain why the majority of patients without NeuGAD had DM, while only 13.3% of patients with DM in our cohort exhibited NeuGAD. The HLA (human leukocyte antigen) class II haplotype DQA1*05:01–DQB1*02:01–DRB1*03:01 was the one most frequently observed in patients with GAD65-ab and NeuGAD. Notably, the DQB1*02:01 allele was significantly more common in patients than in controls (22), while the haplotypes DQA1*05:01-DQB1*02:01-DRB1*03:01 (DQ2-DR3) and DQA1*03:01-DQB1*03:02-DRB1*04 (DQ3-DR4) have been associated with GAD65-ab positive DM. However, the DQ3-DR4 haplotype has not been associated with neurological syndromes GAD65-ab related (23).

### Study limitations

Given the retrospective design of our study, not all variables of interest were systematically assessed, resulting in incomplete data. Therefore, prospective studies with standardized evaluations are warranted to confirm and extend these findings. CSF analysis was performed only in patients with neurological symptoms, and not systematically in all of them. In addition, analytical methods were not uniform, but in cases of diagnostic uncertainty, samples were analyzed at our reference center, Hospital Clínic. Finally, not all patients with high GAD65-ab titers were evaluated by a neurologist trained in epilepsy and/or movement disorders. They were referred once there was a suspicion.

In conclusion, we recommend serum laboratory follow-up in patients with DM and high GAD65-ab titers, and prompt referral to neurology in the presence of neurological symptoms, to allow an earlier diagnosis of NeuGAD. These recommendations are based on observational data with a limited number of events, and prospective studies are needed to confirm these findings and to better define the predictors of neurological involvement in patients with high GAD65-ab titers.

## Data Availability

The raw data supporting the conclusions of this article will be made available by the authors, without undue reservation.
